# Resistance Mechanisms to Chlorpyrifos and F392W Mutation Frequencies in the Acetylcholine Esterase Ace1 Allele of Field Populations of the Tobacco Whitefly, *Bemisia tabaci* in China

**DOI:** 10.1673/031.012.4101

**Published:** 2012-03-24

**Authors:** Ning-ning Zhang, Cai-feng Liu, Fang Yang, Shuang-lin Dong, Zhao-jun Han

**Affiliations:** College of Plant Protection, Education Ministry Key Laboratory of Integrated Management of Crop Diseases and Pests, Nanjing Agricultural University, Nanjing 210095, China

**Keywords:** carbamate insecticides, esterase activity, insensitivity, organophosphate insecticides, resistance selection

## Abstract

The tobacco whitefly B-biotype *Bemisia tabaci* Gennadius (Hemiptera: Aleyrodidae) is a worldwide pest of many crops. In China, chlorpyrifos has been used to control this insect for many years and is still being used despite the fact that some resistance has been reported. To combat resistance and maintain good control efficiency of chlorpyrifos, it is essential to understand resistance mechanisms. A chlorpyrifos resistant tobacco whitefly strain (NJ-R) and a susceptible strain (NJ-S) were derived from a field-collected population in Nanjing, China, and the resistance mechanisms were investigated. More than 30-fold resistance was achieved after selected by chlorpyrifos for 13 generations in the laboratory. However, the resistance dropped significantly to about 18-fold in only 4 generations without selection pressure. Biochemical assays indicated that increased esterase activity was responsible for this resistance, while acetylcholine esterase, glutathione S-transferase, and microsomal-O-demethylase played little or no role. F392W mutations in acel were prevalent in NJ-S and NJ-R strains and 6 field-collected populations of both B and Q-biotype from locations that cover a wide geographical area of China. These findings provide important information about tobacco whitefly chlorpyrifos resistance mechanisms and guidance to combat resistance and optimize use patterns of chlorpyrifos and other organophosphate and carbamate insecticides.

## Introduction

The tobacco whitefly *Bemisia tabaci* Gennadius (Hemiptera: Aleyrodidae) is a small pest with great agricultural importance worldwide (Bellows et al. 1994). The insects not only feed on leaves resulting in delayed growth and even death of the plants (Liu et al. 2007), but also deposit honeydew on leaves that often lead to sooty mold and reduction in photosynthesis (Ghanim et al. 1998). Additionally, the insect is also known to transmit various plant viruses. With increasing acreage of Bt-transgeneic crops such as cotton, which has no resistance to piercing-sucking insects (Ffrench-Constant et al. 2004), the tobacco whitefly problem becomes more serious due to the absence of co-control effects from insecticides previously used to control Lepidopteran pests.

The control of the tobacco whitefly has depended heavily upon synthetic insecticides for decades. As a result, considerable resistance development to a variety of insecticides is very well documented. Understanding of the resistance mechanisms is essential for combating the resistance and improving control efficacy.

In the early days, studies of whitefly insecticide resistance mechanisms were mainly at biochemical and toxicological levels (Hansen and Hodgson 1971; Gunning et al. 1992; Byrne et al. 1995; Gunning et al. 1996; Valles and Woodson 2002). These studies revealed two main mechanisms: reduced penetration and enhanced metabolism of the involved insecticides, the latter playing a more important role. Three groups of enzymes— esterase, glutathione S-transferase (GST), and microsomal-O-demethylase (MFO)—have been proven to be involved in metabolic resistance. For example, Mouches et al. (1986) showed that an esterase gene is responsible for resistance to a variety of organophosphate (OP) insecticides in *Culex* mosquitoes. GST has been showed to play a major role in detoxification of insecticides in mosquitoes (Huang et al. 1998; Vontas et al. 2001; Vontas et al. 2002).

Acetylcholine esterase (AChE, EC 3.1.1.7), a key enzyme in neurotransmission, is the target of organophosphate and carbamate insecticides. Studies with many insect species indicate that resistance to these two classes of insecticides was associated with reduced sensitivity of AChE to insecticides (Mutero et al. 1994b; Walsh et al. 2001; Li and Han 2002; Weill et al. 2003). AChE genes have been cloned in insects the orders Diptera, Hemiptera, Lepidoptera, Hymenoptera, and others (Mutero et al. 1994a; Zhu et al. 1996; Walsh et al. 2001; Vontas et al. 2002; Nabeshima et al. 2003; Cassanelli et al. 2006). Some AChE gene mutations have been confirmed to associate with insect resistance to organophosphate and carbamate insecticides in multiple insect species including *B. tabaci* (Mutero et al. 1994a; Mutero et al. 1994b; Zhu et al. 1996; Walsh et al. 2001; Li and Han 2002; Weill et al. 2003; Alon et al. 2008; Jiang et al. 2009).

Chlorpyrifos has been used to control tobacco whitefly and other insect pests for many years (Pasteur and Sinègre 1978; Milio et al. 1987; Rust and Reierson 1991; Archer 1994; Guides et al. 1996; Liu et al. 2005; Curtis and Pasteur 2009), and is still used to some extent in China and other countries. To combat the resistance problem and prolong the utility of this insecticide, it is essential to understand the resistance mechanisms and resistance levels of field tobacco whitefly populations.

This paper reports the biochemical mechanisms associated with a lab selected chlorpyrifos resistant strain and the frequencies of F392W mutated acel allele in six field populations from locations covering a wide geographic area across China.

## Materials and Methods

### Insects

Tobacco whiteflies were initially collected from cotton fields in the suburb of Nanjing, Jiangsu, China in 2005. They were identified as B-biotype by mtDNA COI sequence analysis (Frohlich et al. 1999). A chlorpyrifos resistant strain was derived from them by exposing part of the population to chlorpyrifos using a spray method at a selection dose around LC_70_. After 26 generations with 22 generations exposed to chlorpyrifos, a resistant strain (NJ-R) was obtained. In the meantime, the rest of the collected population was maintained with no insecticide pressure for 26 generations to obtain a susceptible strain (NJ-S). All insects were reared on caged non-Bt cotton plants at 26 ± 2 °C, 60% RH, and 16:8 L:D photoperiod in the laboratory.

For the F392W mutated acel allele frequencies study, six field populations of tobacco whiteflies were collected from locations in Nanjing, Beijing, Wuhan, Guangdong, Guangxi, and Xijiang provinces in 2010 (Table 1). After being brought back to the lab, live adults were immediately transferred to Eppendorf tubes immersed in liquid nitrogen and stored at -75 °C until used in the experiment. The biotypes of these populations were determined by the same method as above.

### Insecticides and chemical reagents

Technical grade chlorpyrifos (purity > 95%) and triphenyl phosphate (TPP, reagent grade) were purchased from Nantong Insecticide Co., Jiangsu, China; diethyl meleate (DEM, reagent grade), p-Nitro anisole (p-NA), and 1chloro-2,4-dinitrobenzene (CDNB) from Shanghai Chemical Reagent Co. Ltd. (www.richjoint.com); piperonyl butoxide (PBO, reagent grade), NADPH, glutathione (GSH), editic acid (EDTA), 1,4-dithiothreitol (DTT), phenylmethylsulfonyl fluoride (PMSF) phenylthiourea (PTU), and 5,5-Dithiobis (DTNB) disulfide from Sigma-Aldrich (www.sigmaaldrich.com); 1--Naphthyl acetate and fast blue RR salt from Farco Chemical Supplies; and acetylthiocholine iodide (ATChI), bovine serum albumin (BSA), and Triton X-100 from Fluka (www.sigmaaldrich.com). Other chemicals were of analytical quality and purchased from available commercial suppliers.

### Leaf-dip bioassay

The susceptibility of all tobacco whitefly populations used in the experiments was determined using a leaf-dip bioassay adopted from Elbert and Nauen (2000). Briefly, chlorpyrifos stock solutions were prepared in acetone and serially diluted to desired concentrations. Cotton leaf discs (35 mm diameter) were dipped for 10 sec in solutions of insecticides containing 0.2 g L^-1^ Triton X-100 as a non-ionic wetting agent. After the surface was air-dried, a leaf disc was placed onto a bed of agar (1.5 g L^-1^, 10 mm depth) in a plastic dish (35 mm diameter) with the adaxial surface facing downwards. Adult females collected from rearing cages using a pump-powered aspirator were anesthetized with CO_2_, and 25 insects were placed onto each leaf disc. The dishes were sealed with a ventilated lid and stored upside down. Insect mortality was scored after 48 hours. LC_50_ and 95% FL were calculated by Probit regression. The bioassay was conducted at 26 ± 2 °C, 60% RH, and 16:8 L:D photoperiod.

### Synergism assay

Synergism was measured using the above described leaf-dip bioassay. Instead of pure water, 100 mg L^-1^ synergist solutions (TPP, PBO, or DEM) were used as solvents. Control leaf discs were dipped in 100 mg L synergist solutions. Preliminary experiments indicated that 100 mg L^-1^ synergist solutions had no direct toxicity against tobacco whitefly adults. Mortality was scored after 48 hours. LC_50_ values were calculated by Probit regression. Synergism ratio (SR) was calculated as LC_50_ of insecticide alone/LC_50_ of insecticide with synergist.

### Detoxification enzyme activity assays

**Esterase.** 40 adults from NJ-S or NJ-R were homogenized in an 800 µL ice-cold sodium phosphate buffer (0.2 M, pH 7.8) at 4 °C. The homogenate was then centrifuged at 10,000 g for 20 min. The supernatant was used as an enzyme source. Esterase activity was measured according to Han et al. (1998) by adding 80 µL enzyme source into 120 µL 0.2 M substrate solution containing Fast Blue RR salt in sodium phosphate buffer (0.2 M, pH 7.8) and 1 mM 1-naphthylacetate. Reactions were read by a Versamax kinetic microplate reader (Molecular Devices, LLC, www.moleculardevices.com) recording at 20 sec intervals for 7 min at 450 nm and 27 °C.

**MFO.** 20 mg of adult tobacco whitefly (mixed sexes) from NJ-S or NJ-R strains was homogenized with 500 µL ice-cold sodium phosphate buffer (0.2 M, pH 7.8, contained EDTA, DTT, PTU, PMSF, and glycerol). The homogenate was then centrifuged at 13,000 g for 30 min at 4 °C. After filtered with glass wool, the supernatant was re-centrifuged at 13,000 g for 20 min, and the supernatant was used as an enzyme source. Enzyme activity was measured by mixing 100 µL enzyme source with 20 µL 2 mM substrate solution (pNA) and 10 µL 9.6 mM NADPH. The reaction was read by the microplate reader at 20 sec intervals for 15 min at 405 nm and 27 ° C (Hansen and Hodgson 1971).

**GSTs.** Enzyme source was prepared from 80 adults (mixed sexes) in the same way as MFO with no filtration process after the first centrifugation. Activity was measured by mixing 100 µL enzyme source with 20 µL 1.2 mM substrate solution CDNB and 100 µL 6 mM GSH. The reaction was read by the c microplate reader at 20 sec intervals for 10 min at 340 nm and 27 °C (Oppenoorth et al. 1979).

All measurement was done in five replicates. SOFTmax software was used to fit kinetic plots by linear regression. Enzyme activity (Vmax) was expressed in mOD/min.

### AChE kinetics parameters and Ki assay

60 adults (mixed sexes) from NJ-S or NJ-R strain were homogenized with 500 µL icecold sodium phosphate buffer (0.2 M, pH 7.6, contained 0.05% Triton X-100). The homogenate was centrifuged at 13,000 g for 10 min at 4 °C, and the supernatant was centrifuged again at 13,000 g for 20 min. The supernatant that resulted from the second centrifugation served as the enzyme source.

AChE kinetics parameters were measured according to the method by Li and Han (2002). The reaction system in volume of 100 µL contained substrate analogue acetylthiocholine (ATChI) at final concentrations ranging from 31.25 µM to 500 µM and DTNB (in buffer solution) at a final concentration of 450 µM. AChE activity was measured at 30 sec interval for 30 min by the microplate reader at 405 nm and 25 °C. Double reciprocal method was used to obtain Km and Vm (expressed in mOD/min).

Ki of AChE was determined according to the method reported by Moores et al. (1996). The reaction solution was prepared by mixing 100 µL of enzyme source and 100 µL chlorpyrifos-methyl (50 ppm) at 25 °C. An aliquot of 20 µL reaction solution was taken out every other 20 sec, mixed with 80 µL 0.02 mol L^-1^ sodium phosphate buffer (pH 7.0), 100 µL DTNB (450 µM), and 100 µL ATChI (1.5 mM). The reaction was subsequently read with the microplate reader at intervals of 30 sec for 30 min at 405 nm and 25 °C. All measurements were done in five replicates. SOFTmax software was used to fit kinetic plots by linear regression.

Total protein content of all used enzyme sources was determined by Coomassie brilliant blue method using bovine serum albumin as a standard (Bradford 1976).

### Cloning and analysis of acel gene fragments

Total RNA was extracted using TRIzol® reagent (Invitrogen, www.invitrogen.com) from 100 adults according to manufacturer instructions. First-strand cDNA was synthesized from the total RNA using ThermoScriptTM reverse transcriptase (Invitrogen). PCR for cloning BT-acel fragments was performed by LA Taq polymerase (TaKaRa Co. www.takarabio.com) and 2 × GC Buffer II (TaKaRa Co.) with following parameters: 94 °C for 2 min followed by 40 cycles at 94 °C for 30 sec, 60 °C for 30 sec, 72 °C for 2 min, and one additional cycle at 72 °C for 10 min. The sense and anti sense primers used for this PCR were designed from the reported BT-acel sequence (ncbi nucleotide: EF675188.1), which are F5′→3′ ATGGACTTCGATCACCTCCCTCTCA and W5′→3′ CGGTGACGAATGACTGGATAAT, respectively. PCR products were separated by agarose gel electrophoresis, purified with AxyPrepTM DNA Gel Extraction Kit (Axygen Biosciences, www.axygenbio.com), and then cloned into pGEM-T easy vector (Promega, www.promega.com). The ligation reactions were used for transformations with the DH5a competent cells. Positive clones were screened with blue/white and standard ampicillin selection. Recombinant plasmids were fully sequenced by Invitrogen.

For PCR of individual whiteflies, the same method and protocol were used with the following modifications. All reagents used in RNA extraction were in half amounts. PCR conditions were 94 °C for 2 min followed by 40 cycles at 94 °C for 30 sec, 60 °C for 30 sec, 72 °C for 40 sec, and one additional cycle at 72 °C for 10 min, with two primers of F5′→3′ CCTTCCTGGACGAGATGCC and R5′→3′ CGCCGCACGATGAAGTTGT.

### PCR-RFLP assay

The PCR-RFLP assay was adopted from Tsagkarakou et al. (2009). Genomic DNA (gDNA) was extracted from individual adults by DNeasy Blood and Tissue Kit (Qiagen, Germany). The primer pairs used in the PCR were Test-F (5′-TAGGGATCTGCGACTTCCC-3′) and Test-R (5′-GTTCAGCCAGTCCGTGTACT-3′), by which a 287 bp fragment was amplified. This fragment was fully digested with the restriction enzyme Bsrl (MBI Fermentas, www.fermentas.com). As susceptible acel allele contains two sites and resistant acel allele contains three sites for restriction endonuclease Bsrl, digestion of the PCR product with Bsrl yields a restriction pattern of three fragments (201, 79, and 7 bp) for the susceptible acel allele and four fragments (140, 61, 79, and 7 bp) for the resistant acel allele.

Amplifications were performed with approximately 20 ng gDNA in 10x Ex Taq reaction buffer (TaKaRa) with 4 µL MgCl_2_ at a final concentration of 25 mM, 10 µM of each primer (Test-F and Test-R), 1 µL , 2.5 mM dNTP 41 µL and 1.25 U Ex taq. PCR cycling conditions were 94 °C for 5 min, 35 cycles of 95 °C for 15 sec, 52 °C for 30 sec, 72 °C for 40 sec, followed by 72 °C for 10 min. The amplification product was incubated for three hours in a reaction buffer (TaKaRa) with 5 U Bsrl. Digested products were electrophoresed using a 3% (w/v) agarose gel. The 7 bp fragments could not be detected, as they were too small to be visualized by electrophoresis. Sixteen to eighteen individuals were examined for each of the field populations of the tobacco whitefly.

## Results

### NJ-R strain establishment

**Figure 1.**
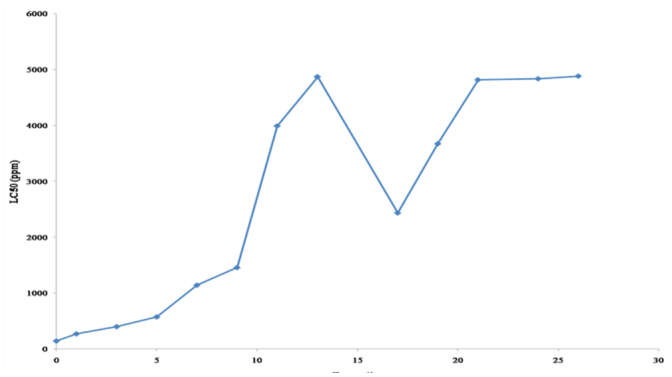
Resistance development of NJ-R *Bemisia tabaci* strain selected with chlorpyrifos in a dose of around LC_70_ in laboratory. LC_50_S were examined every two or three generations. The selection by chlorpyrifos was stopped from the 13^th^ to 16^th^ generation, and then restored at the 26^th^ generation. High quality figures are available online.

To investigate the development process of the tobacco whitefly resistance to chlorpyrifos, a resistant strain (NJ-R strain) was selected from a field population in the laboratory (Figure 1). During the course of resistance selection, the LC_50_ increased slowly but steadily in 1^st^ to 9^th^ generations (from 143.90 ppm to 1458.30 ppm), and afterwards LC_50_ increased in a much faster pace to reach 4874.10 ppm at the 13^th^ generation. At this point, the selection was stopped for the following four generations, and as a result, the LC_50_ declined sharply to about 2500 ppm measured in 17^th^ generation. However, with additional selection the LC_50_ was recovered to 4818.02 ppm at the 21^st^ generation. Continuing selection in the 21^st^ to 26^th^ generation did not result in increasing LC_50_, but maintained a value around 4800 ppm. This field collected tobacco whitefly population had a 33.94-fold chlorpyrifos resistance based on the LC_50_ ratio after facing selections in 22 of the 26 generations. In the meantime, from the part of the same population used for resistance selection, a relative susceptible strain (NJ-S strain) was obtained by maintaining it without exposure to any insecticide for 26 generations. These selected resistant and susceptible strains were further used to explore the resistance mechanisms.

### Metabolic enzyme activity of NJ-R and NJS strains

To understand the metabolic resistant mechanism involved in the chlorpyrifos resistance of NJ-R strain, the activities of three major metabolic enzymes were measured and compared between NJ-R and NJ-S strains (Table 2). The results indicated that the esterase activity of NJ-R was significantly higher than that of NJ-S (increased 1.53-fold). However, no significant difference was found in GSTs and MFO activities between NJ-R and NJ-S strains.

### Synergism of TPP, DEM and PBO

TPP, DEM, and PBO are specific inhibitors of esterase, GSTs, and MFO, respectively. To confirm the results of enzyme activity measurement, the synergisms of TPP, PBO, and DEM with GSTs and MFO activities on NJ-R and NJ-S strains were determined. The results revealed that TPP had an obvious synergism to chlorpyrifos on both strains, with the synergism ratios of 4.46 and 2.43 in NJ-R and NJ-S strains, respectively. However, DEM and PBO had no significant synergism to chlorpyrifos in both strains (Table 3). The tests confirmed that enhanced esterase activity is at least partially responsible for the observed chlorpyrifos resistance.

**Figure 2.**
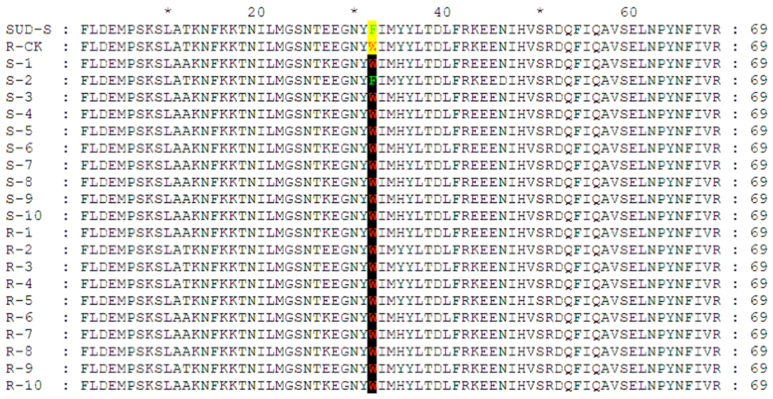
Examination of F392W point mutation in 10 NJ-S *Bemisia tabaci* (from S-1 to S-10) and 10 NJ-R tobacco whiteflies (from R-1 to R-10) by cloning a 211 bp-acel gene fragment. SUD-S (ncbi protein: ABV45413.1) and R-CK (ncbi protein: ABV45421.1) served as susceptible and resistant references. All 10 NJ-R individuals and 9 out of 10 NJ-S individuals carried the F392W mutation. High quality figures are available online.

### Inhibition kinetics of AChE

To explore the target mechanisms of this chlorpyrifos resistance in NJ-R strain, the Km, Vm, and Ki of AChE preparations from both NJ-R and NJ-S strains are shown in Table 4. There was no significant difference in Km, Vm, and Ki between NJ-R and NJ-S strains. The results suggested that AChE was not involved in this chlorpyrifos resistance of the NJ-R strain relative to the NJ-S strain of the tobacco whitefly.

### Frequencies of F392W mutation in NJ-R and NJ-S

Since F392W mutation in acel gene was already reported to be associated with OP resistance in the tobacco whitefly (Alon et al. 2008), the F392W mutation frequencies in NJR and NJ-S strains were determined. Acel fragments were cloned using cDNA template from a pooled sample of 100 insects of NJ-R and NJ-S strains, respectively. Three clones of PCR products from each strain were randomly chosen to be sequenced. Unexpectedly, the reported F392W mutation was present in all six sequenced clones, and no other mutation was found in the acel-1895 bp fragments.

To confirm this result, a PCR assay of individual tobacco whitefly was conducted using primers that are able to amplify a 221 bp fragment across the F392W mutation site in acel specifically. One out of 10 NJ-S individuals showed the sensitive genotype (F392) and the other nine had the F392W mutation, whereas all 10 individuals from NJR strain displayed the F392W mutation (Figure 2). The results indicated that F392W mutation was not responsible for the chlorpyrifos susceptibility difference between NJ-S and NJ-R stains, and that both NJ-S and NJ-R strains had a similar level of target resistance.

### Frequencies of F392W mutated acel allele in field population

As it was reported that the F392W mutation in acel gene resulted in the OP resistance of the target insensitivity, frequencies of F392W mutation in acel gene in 6 geographically distinct populations across China were investigated by the PCR-RFLP assay. High frequencies of the mutation were found in all 6 field populations (Table 5). All 18 tested whiteflies from the Beijing population were mutant homozygotes, and most individuals (> 88%) from the other 5 populations also were mutant homozygotes. In addition, 2 of the 16 whiteflies from Guangdong and Xinjiang population were mutant heterozygotes. Among the tested individuals, only 4 individuals (two from Nanjing population, one from Wuhan population, and one from Guangxi population) were wild homozygotes.

## Discussion

This study showed that B-biotype tobacco whitefly can develop chlorpyrifos resistance (NJ-R) under continuous selection pressure. The 34-fold laboratory selected resistance involves metabolic mechanisms that confer resistance to a certain extent as the LC_50_ reached a plateau in the selection process. The substantial drop of LC_50_ from the plateau level (33.9 fold) to the level of 16.9 fold in only 4 generations without selection pressure suggested that such metabolic resistance has a high fitness cost. This high fitness cost of the metabolic resistance to chlorpyrifos can partly explain that chlorpyrifos still retains a relatively higher control efficacy against tobacco whitefly after decades of use in the field. Tobacco whitefly control in China always employs several insecticides with different modes of action, and chlorpyrifos is rarely applied consecutively more than 5 times. This practice curbs the development of the resistance and should be continued.

Metabolic enzyme activity analysis showed that esterase plays a major role in the resistance as no significant difference in GSTs and MFO activities between NJ-S and NJ-R stains was found. Synergism experiments delivered the same conclusion as only TPP resulted in a higher synergism ratio (SR) for NJ-R. This result agrees with Alon et al. (2008), but differs from abamectin resistance in tobacco whitefly and *T. urticae*, where detoxification of MFO and GSTs was indicated as a key factor (Stumpf and Nauen 2002; Wang and Wu 2007). This is not necessarily unexpected, as insecticides of different action modes often induce resistance with different mechanism even in same insect species.

As the target of OPs and carbarmates, insensible OP site mutations of AChE have been identified in insects (Mutero et al. 1994a, 1994b; Zhu et al. 1996; Walsh et al. 2001; Vontas et al. 2002; Nabeshima et al. 2003; Cassanelli et al. 2006). Specific to tobacco whitefly, an F392W mutation was shown to be responsible for OPs resistance (Alon et al. 2008). However, AChE kinetic parameters in our study showed no significant difference between NJ-R and NJ-S strains. This result suggests that resistance in NJ-R was not due to AChE site mutations. However, to our surprise, sequence analysis of acel-1895 bp fragments from pooled samples of NJ-R or NJ-S showed consistent F392W mutations compared to the wild type SUS-S strain (ncbi protein: ABV45413.1). A further analysis with a single tobacco whitefly showed that all 10 tested NJ-R individuals and nine out of 10 NJ-S individuals possessed the F392W mutation. Therefore, the NJ-S strain used in our study was a 'susceptible' strain already carrying the target site resistance. This is indirectly supported by the fact that chlorpyrifos LC_50_ of SUD-S strain in Alon et al. (2008) was much lower (4.57 ppm) compared to NJ-S stain (137.55 ppm) in our study. The results indicated that resistance can have multiple mechanisms for any give insecticide and insect species. Conducting a complete investigation in understanding resistance mechanisms, as shown in this study, is a good method for future research.

The investigation of mutant acel gene frequencies in field populations revealed that high frequencies (88–100%) of F392W mutant acel allele were found in all six field populations of different biotypes from a wide geographic area of China, and most individuals (92%) were resistant homozygotes. This result indicated that F392W mutant acel in *B. tabaci* associated to OP, and carbamate insecticide resistance is widespread; this should be taken into consideration when designing insecticide rotation programs for whitefly management.

As an invasive pest, B-biotype *B. tabaci* was first introduced into China at the end of 1990s. By 2003, it had rapidly spread into 25 provinces and become the dominating biotype. Fast and strong development of insecticide resistance was one of the key factors contributing to this successful invasion and rapid spreading (Liu et al. 2008). Byrne and Devonshire (1993) reported that a large proportion of B-biotype whiteflies in United Kingdom carried insensitive AChE capable of conferring extremely high resistance to OP and carbamate insecticides. The fact that a similar high level of acel mutation frequencies (88–100%) were detected in all six geographically different populations leads us to speculate that this mutation was already present at the time of invasion, and the insecticide selection pressure after invasion had little effect on the mutation frequency because the insecticide use patterns as well as invasion time were different among the six locations where the tested populations were collected. Unfortunately, no baseline data (at the time of invasion) on the mutated acel frequency in tobacco whitefly field populations (B- or Q-biotype) are available in China. This speculation remains a hypothesis waiting to be accepted or rejected.

**Table 1.**
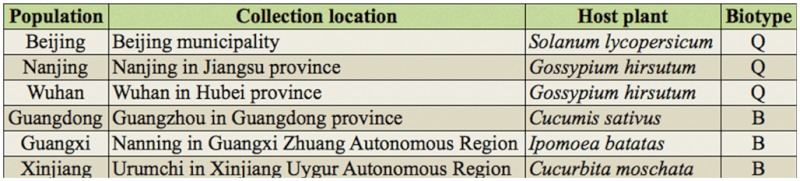
Collection locations, host plants, and biotypes of *Bemisia tabaci* field populations.

**Table 2.**
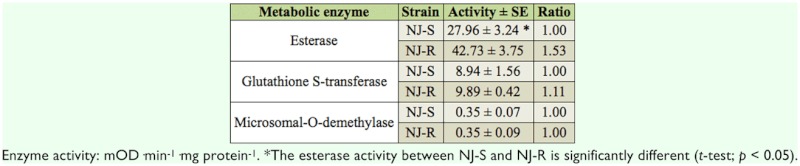
Metabolic enzyme activities of NJ-R and NJ-S strains.

**Table 3.**
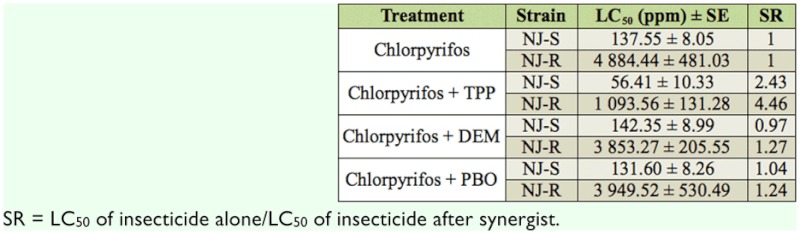
Synergism of TPP, PBO, and DEM on chlorpyrifos in NJ-R and NJ-S strains of *Bemisia tabaci*.

**Table 4.**
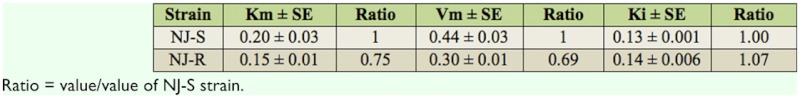
Kinetic parameters and Ki values of AChE from NJ-R and NJ-S strains of *Bemisia tabaci*.

**Table 5.**
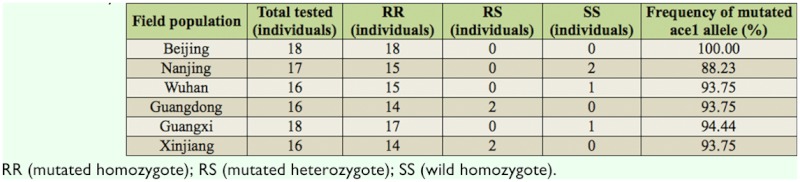
Genotypes and F392W mutation frequencies in acel of six field *Bemisia tabaci* populations from China, detected by the PCR-RFLP assay.

## Abbreviations

AChE,: acetylcholine esterase;
DEM,: diethyl meleate;
GST,: glutathione S-transferase;
MFO: microsomal-O-demethylase;
NJ-R,: Nanjing Jiangsu resistant *B. tabaci* strain;
NJ-S,: Nanjing Jiangsu susceptible *B. tabaci* strain;
OP,: organophosphorus;
PBO,: piperonyl butoxide;
TPP,: triphenyl phosphate
